# The research software engineering (RSE) survey dataset (2016–2022): A longitudinal and international resource for studying the research software workforce

**DOI:** 10.1016/j.dib.2026.112873

**Published:** 2026-05-18

**Authors:** Wioleta Kijewska, Heather S. Packer, Simon Hettrick

**Affiliations:** a University of Southampton, University Road, Southampton SO17 1BJ, United Kingdom; b The Software Sustainability Institute, EPCC, University of Edinburgh, The Bayes Centre, 47 Potterrow, Edinburgh EH8 9BT, UK

**Keywords:** International survey data, Longitudinal dataset, Research software workforce, Employment and career pathways, Software development practices, Software sustainability

## Abstract

The Research Software Engineering (RSE) Survey dataset contains longitudinal survey data collected by the Software Sustainability Institute between 2016 and 2022. The survey was initially conducted in the United Kingdom and expanded in subsequent years to include multiple countries. From 2018 onward, a single international instrument was used across participating countries, with only minor contextual adaptations to maintain comparability. Participation was voluntary and open to individuals who self-identified as Research Software Engineers or as performing research-software-related work, irrespective of formal job title.

The dataset includes anonymized responses covering demographics, employment conditions, coding practices, training and collaboration, publication contributions, sustainability practices, professional networks, and job satisfaction. The **RSE Survey Report**, an interactive dashboard (W. Kijewska, M. Donnay, H. S. Packer, S. Hettrick, RSE survey report, [software] (2026). URL https://rse-survey.soton.ac.uk/superset/dashboard/RSE_survey/), also enables exploration of trends, cross-country comparisons, and changes across survey waves.

The dataset is openly licensed and supported by publicly available analysis code, enabling reproducible workflows. The longitudinal and international design supports reuse for cross-sectional and temporal analyses, workforce comparisons, and integration with external datasets such as national workforce statistics or research output indicators. The dataset will be expanded with results from future surveys, including the 2026 survey that is pending completion.

Specifications Table

**Table:** 

Subject	Computer Sciences
Specific subject area	Longitudinal and international record on employment conditions, work and professional activities, and recognition of Research Software Engineers.
Type of data	Tables Raw and Processed
Data collection	Data were collected by survey aimed at people involved in creating software for research by Research Software Engineering Organisations in UK (2016, 2017, 2018, 2022), Germany, USA, Canada, the Netherlands, South Africa (all in 2017, 2018, 2022), Australia, New Zealand (both in 2018 and 2022), and other countries on a smaller scale (2018 and 2022). Respondents were recruited by distributing email invitations within their organisations and by providing a link to the survey on the Software Sustainability Institute's website.
Data source location	UK (2016, 2017, 2018, 2022), Germany, USA, Canada, The Netherlands, South Africa (all in 2017, 2018, 2022), Australia, New Zealand (both in 2018 and 2022), and other countries on a smaller scale (2018 and 2022).
Data accessibility	Repository name: Zenodo Data identification number: https://doi.org/10.5281/zenodo.20072506 Direct URL to data: https://github.com/softwaresaved/RSE_survey_longitudinal
Related research article	None

## 1 Value of the Data

• This dataset [1] provides a comprehensive, longitudinal record of the demographics, employment conditions, working practices, and professional activities of RSEs, collected consistently since 2016 across an expanding set of countries. This multi‑year and international scope enables cross‑country and temporal comparisons of RSE roles, career pathways, job satisfaction, and training activity.
• The dataset has been used by the Software Sustainability Institute to describe the employment structures, coding patterns, training activities, and community characteristics of the RSE, based on multiple surveys from 2016 [2], 2017 [3], 2018 [4], and 2022 [5]. These analyses demonstrate the dataset’s utility as an evidence base for understanding RSE workforce structures and research software practice.
• The survey’s breadth and methodological consistency make it a valuable resource for examining structural characteristics of the RSE profession and for informing policy, organisational practices, and community initiatives related to fairness and inclusion in research software roles. The associated analysis [6] identifies areas of under-representation and supports the development of targeted, evidence-based interventions.
• The data is anonymized, openly licensed, and supported by publicly available analysis code in GitHub repositories, enabling reproducible research workflows and facilitating reuse by policymakers, institutions, and researchers.
• The survey structure allows integration with external datasets, such as national workforce statistics or research output indicators, supporting research on research capacity, investment planning, and the professionalization of research software roles.

## 2 Background

The dataset compiles responses from the RSE Surveys conducted between 2016 and 2022. The series began in 2016 in the UK to capture information about individuals developing software used in research, at a time when the professional identity of Research Software Engineers was still emerging within academia. The survey expanded in 2017 to include Canada, Germany, the Netherlands, South Africa, and the United States. Countries joined both to understand the roles, activities, and working conditions of RSEs in their own research systems and to raise awareness of Research Software Engineering by showing that the community was active and growing internationally.

By 2018, the survey was delivered as a single international questionnaire, with only minor adjustments for national contexts. Subsequent waves broadened global coverage and collected anonymised, harmonised data on demographics, employment contexts, coding practices, training, collaboration, publication contributions, sustainability practices, and professional networks.

Although not included in this dataset, a new iteration of the International RSE Survey launched in 2026 continues this multi-year effort, supporting national associations, funders, and policymakers in understanding the needs of the global RSE community and introducing updated questions on the use of AI tooling in research software.

## 3 Data Description

The dataset contains files for each year the survey was conducted following the below naming pattern, for every folder named by the year of the survey it relates to (2016, 2017, 2018, and 2022):

**{year}_cols.csv** Overview of questions used for each year of the survey together with the corresponding column name and options associated with the question (for multiple-choice questions). The questions cover a wide range of topics related to the position of a Research Software Engineer and some demographic information.

**{year}_tf.csv** Raw survey data where Yes/No questions are saved in a Boolean True/False format. Multiple-choice questions are stored in a wide format, where each option is in a separate column. The number of columns varies between 68 (for 2016 data) and 368 (2017 data).

**{year}_{question code}.csv** Files where multiple-choice and free-text (of the type ``Which 3 skills would you like to acquire?'') questions are converted to long format in a single column (multiple entries for the same response ID).

**{year}_{sensitive data}.csv** Files with sensitive data (disability, ethnicity, gender, salary) disconnected from the main dataset. Every entry retained information about the country (question “socio1_0'') of the participant. Each file is coupled with its own “_cols.csv'' file containing the column names and options. Ethnicity data is also reformatted into a long table in the “{year}_ethnicity_long.csv'' file.

The dataset also contains folder 'questionnaires' where full questionnaires for every year are included. Additionally, the dataset contains the “2017_merging_list.csv” which outlines which variables from the 2017 survey have been merged based on the similarity of their questions. Data from other survey waves did not require variable merging.

In addition to downloadable, openly licensed data files, the longitudinal survey results are also accessible through an interactive dashboard [7], which enables users to explore trends, compare countries, and examine changes across survey waves. The dashboard is intended to facilitate exploratory analysis and support the development of research hypotheses prior to downloading the dataset for further investigation.

## 4 Experimental Design, Materials and Methods

This section describes the design of the survey instruments, the procedures used for participant recruitment and data collection, and the methods applied to curate, harmonise, and prepare the resulting datasets for reuse. The intent is to document the methodological decisions that shaped the structure and content of the dataset, to support transparency, reproducibility, and longitudinal cross-sectional comparison across survey waves and participating countries.

### 4.1 Survey design and scope

Established in 2016, the survey later became a multi‑national effort, with 2018 and 2022 marking the first fully international waves. The survey focused on collecting comparable data from academic and government research software developers, with industry participants welcome but not included in the design scope.

*The core survey framework comprises ten thematic subject areas:*

• Demographics,
• Coding activity,
• Employment,
• Current contract,
• Previous employment,
• Collaboration and training,
• Publications,
• Sustainability and tools,
• Job satisfaction, and
• Professional networks.

Although minor national adaptations were introduced, which were limited to phrasing, job titles, and additional questions requested by local RSE societies and reflecting national contexts; the overall structure was maintained to support longitudinal analysis. The set of questions was expanded and revised over time, and each wave of the survey underwent pilot testing and a test deployment prior to launch.

The 2016 survey was available only in English, as it was focused on respondents from the UK, and data were collected using iSurvey [8], provided and maintained by the University of Southampton. From 2017 onward, respondents could select one of four survey languages irrespective of country of work. LimeSurvey’s [9] multilingual capabilities enabled delivery in English, French, German, and Spanish. All translations were reviewed by native speakers. The survey employed branching and conditional logic to ensure that respondents were only shown questions relevant to their circumstances.

The International RSE Surveys are developed by international committees representing a broad cross‑section of the global Research Software Engineering community. These committees collaboratively design, review, and refine the survey instruments to ensure relevance across national contexts and to support meaningful cross‑country comparisons. For the 2022 survey, contributors from Europe, North America, South America, Africa, and Asia shaped the questionnaire’s content and structure. Similarly, the 2017 and 2018 survey cycles were created by international teams that worked to harmonise question sets and coordinate the transition toward a unified global survey. No AI tools were used in the development of questionnaires for any wave of the survey.

Participant recruitment was coordinated by local RSE organisations. This involved advertising the survey on their websites (including the Software Sustainability Institute’s website) and distributing an open‑access public link via email to members of each organisation. Eligible respondents were those involved in the creation of software for research, whether by writing software or by leading projects or groups engaged in research software development. Further detail on participating countries and respondent demographics is provided in Tables 1, 2, and 3. No probability-based sampling or other formal sampling frame was involved in the collection of responses. Participation was purely voluntary and based on self-identification. We are unable to compute the response rates due to the use of open distribution channels.

**Table 1:** Dataset statistics.

	2016	2017	2018	2022
Number of columns in main file	68	368	288	298
Number of responses	273	951	997	1003

**Table 2:** Top 10 countries with the largest number of participants.

Country	2016	2017	2018	2022
United Kingdom	273	253	237	176
Germany	-	325	333	258
United States	-	164	147	161
Netherlands	-	77	54	66
Canada	-	110	12	11
Australia	-	-	99	33
France	-	-	5	77
New Zealand	-	-	37	28
Finland	-	-	-	58
South Africa	-	22	23	2

**Table 3:** Demographics of participants for all waves of RSE Survey.

*Variable*		*N*	*Percent*
**Age Group (years)**			
	*18 to 24*	*27*	*1.7*
	*25 to 34*	*541*	*34.7*
	*35 to 44*	*631*	*40.5*
	*45 to 54*	*253*	*16.2*
	*55 to 64*	*88*	*5.6*
	*65 or older*	*7*	*0.4*
	*Prefer not to say*	*12*	*0.8*
**Gender**			
	*Male*	*1973*	*82.5*
	*Female*	*333*	*13.9*
	*Prefer not to say*	*70*	*2.9*
	*Prefer to self-describe*	*9*	*0.4*
	*Other*	*6*	*0.3*
**Education Level**			
	*Doctoral or equivalent (ISCED 8)*	*1730*	*57.12*
	*Masters or equivalent (ISCED 7)*	*945*	*31.13*
	*Bachelors or equivalent (ISCED 6)*	*313*	*10.31*
	*Pre-Bachelors (up to ISCED 5)*	*23*	*0.76*
	*Other*	*21*	*0.69*
***Working Pattern***			
	*Full time*	*2740*	*88.5*
	*Part-time*	*326*	*10.5*
	*Contract*	*8*	*0.3*
	*Graduate student*	*4*	*0.1*
	*Co-op placement/internship*	*2*	*0.1*
	*Self-employed*	*1*	*0.03*
	*Other*	*4*	*0.1*
	*Prefer not to say*	*12*	*0.4*

### 4.2 Data collection procedures

Each survey wave was administered online using either iSurvey [8] (in 2016) or LimeSurvey [9] (2017 and later) and remained open for a defined period appropriate to the data‑collection cycle as specified in Table 4. Surveys were delivered through standard web browsers that allow JavaScript and cookies, including browsers on mobile phones. The survey‑status cookie was not enabled, meaning participants could not resume the survey from where they had left off.

**Table 4:** Start and end dates of each survey wave.

Survey wave	Country	Start of survey	End of survey
**2016**		March 2016	May 2016
**2017**	Canada	Late 2017	Early 2018
	Germany	October 2017	January 2018
	Netherlands	November 2017	December 2017
	South Africa	November 2017	January 2018
	United Kingdom	April 2017	June 2017
	United States	November 2017	January 2018
**2018**		September 2018	January 2019
**2022**		November 2021	March 2022

Participation was voluntary and open to anyone who self‑identified as a Research Software Engineer or carried out research‑software‑related work, regardless of their formal job title. Each participant was presented a unique ID number at the end of the survey, to be able to identify their submission if they wanted to contact the organisers about it at a later date. No other direct identification data was collected. Multiple submissions were not prevented but they were not expected.

Respondents accessed the survey through a public link distributed by local RSE organisations. Before beginning the survey, participants were shown a Participant Information Sheet, providing introductory information describing the survey’s purpose. Participation was fully anonymous and no location metadata were stored.

In line with the ethics procedures in place for each wave, consent was obtained through an on‑screen confirmation: earlier surveys required respondents to click an explicit “I accept” button, while later surveys asked participants to confirm their willingness to take part by clicking the “Next” button beneath the information sheet, as stated in the consent text.

From 2018 onward, respondents were able to choose the survey language irrespective of the country where they were based and could be changed mid-survey as all question sets were identical across all available languages.

The survey captured self‑reported information on respondents’ work activities, skills, contractual arrangements, disciplinary backgrounds, training practices, and workplace contexts. Branching and conditional logic was used to ensure that respondents only received questions relevant to their circumstances. In total, up to 25 questions in each survey only appeared after the required answers to related questions were chosen. Some of those were country-specific.

Since 2018, participants completed a version of the survey linked to their country of work. Most questions were non‑mandatory, allowing participants to skip items as needed. Some short free-text boxes were included in the 2017 survey, with a character limit of 255. All other free-text questions had an answer limit of 65 000 characters without auto-complete.

After the survey closed, data were exported from the survey platforms into .csv files for all countries. Filling in the whole survey took on average between 10 minutes in 2016 and up to 30 minutes for later surveys.

### 4.3 Data processing and curation

Survey responses were collated from their corresponding repositories ([2] for 2016, [3] for 2017, [4] for 2018, and [5] for 2022), cleaned, and curated into structured datasets to support reuse and longitudinal analysis. No deduplication was required, as all submissions were treated as unique. Incomplete surveys were excluded from the final datasets. Missing values have been recorded as NaN (Not a Number), following Pandas conventions. No imputation was applied, and inconsistencies and outliers have been preserved in their raw form. This is to ensure that downstream users can apply their own domain-specific cleaning rules following assumptions appropriate to their research question.

Variable names were standardised to a common schema across survey years to support cross-wave comparison. Any new questions were given a new code at the survey creation stage. While the 2017 question codes differed between participating countries, their text was compared and questions with the same meaning were merged (see “2017_merging_list.csv” file for details). Any questions that could have been interpreted differently were kept in the dataset in their original form.

Yes/No questions were recoded into Boolean True/False values. Text fields were normalised by converting to lowercase and trimming whitespace. Each survey contained multiple-choice questions originally stored in a wide format, where each option had its own column; these were reshaped into long-format tables stored in individual files. Free-text list-type responses (e.g., conference names) were tokenised using regular expressions and stored in long format. Attitude and perception-type questions were asked using the Likert format [10] using 5-level scales, while questions relating to the proportion of time used a 10-level scale to reflect decimal and percentage-based thinking.

Sensitive data, such as disability, ethnicity, gender, or salary information, were separated from the main table to minimise re-identification risk, while retaining a link to respondents’ chosen country of work, without any respondent ID or other primary keys, as per the ethics approval. The ethnicity question was included only in the UK and US surveys, using country-specific categorisation. The salary question asked only about the salary range, specified by the country's RSE organisation's representative and updated for each survey wave as needed.

Data from the 2017 country-specific surveys were collated into a single dataset. Wherever possible, equivalent questions were combined into harmonised columns, while unmatched questions were retained in their own fields. Since 2018, respondents have been able to choose their country of work from a drop-down list of names according to ISO-3166-2.

All datasets were versioned to support transparent tracking of changes over time. The data was processed using bespoke scripts written in Python 3, with the use of Pandas and regular expressions.

For ease of further analysis, multiple-choice questions have been transformed into a long format using the “melt” function from Pandas, keeping the “row_id” variable as primary key linking back to the main file. Comma-separated questions have undergone a similar procedure using the split and explode functions. All of those questions are kept in their original wide format in the main “{year}_tf.csv” files.

The full dataset was visualised in an interactive dashboard created in Apache Superset 4.1.4 and published for public view [7]. The data was additionally processed in MySQL for the purpose of the dashboard and divided into smaller datasets for ease of visualisation. Query for each visualisation can be viewed directly from the dashboard interface.

**Fig. 1:**
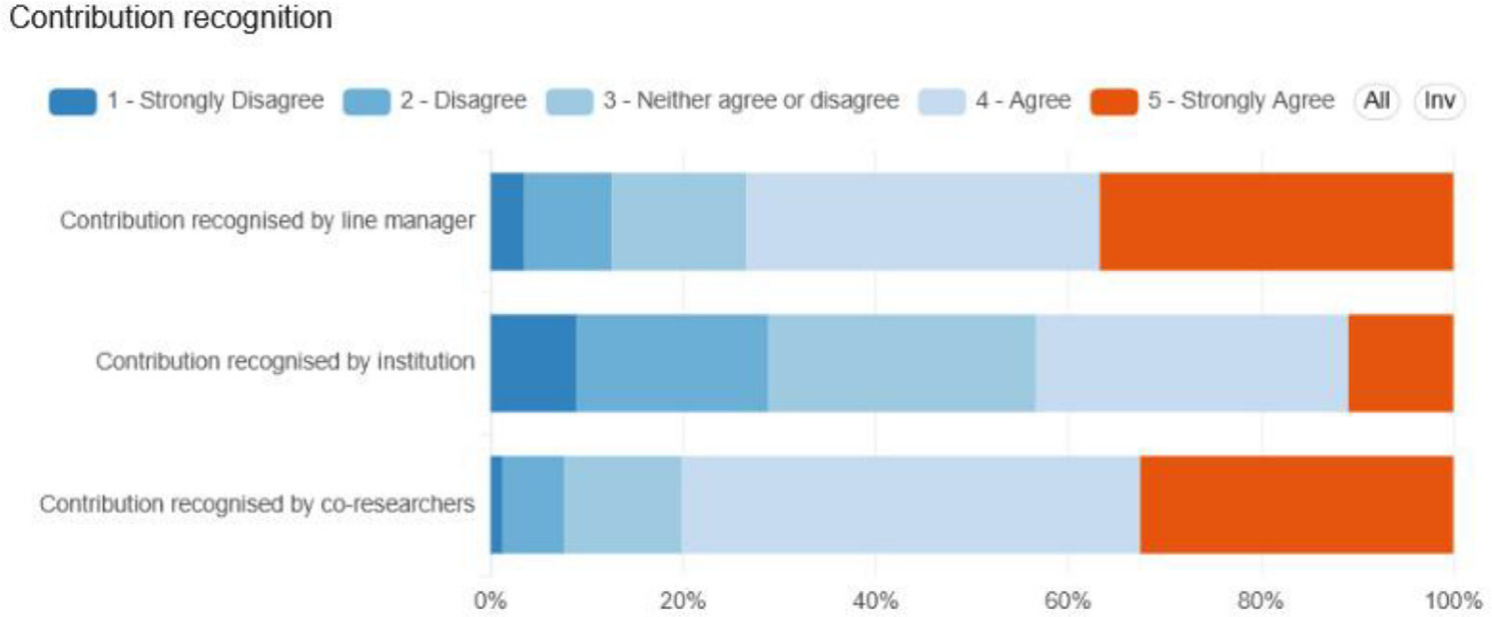
Answers to the question ``I feel that my contribution to research is recognised by my institution/line manager/co-researchers.''. [7].

**Fig. 2:**
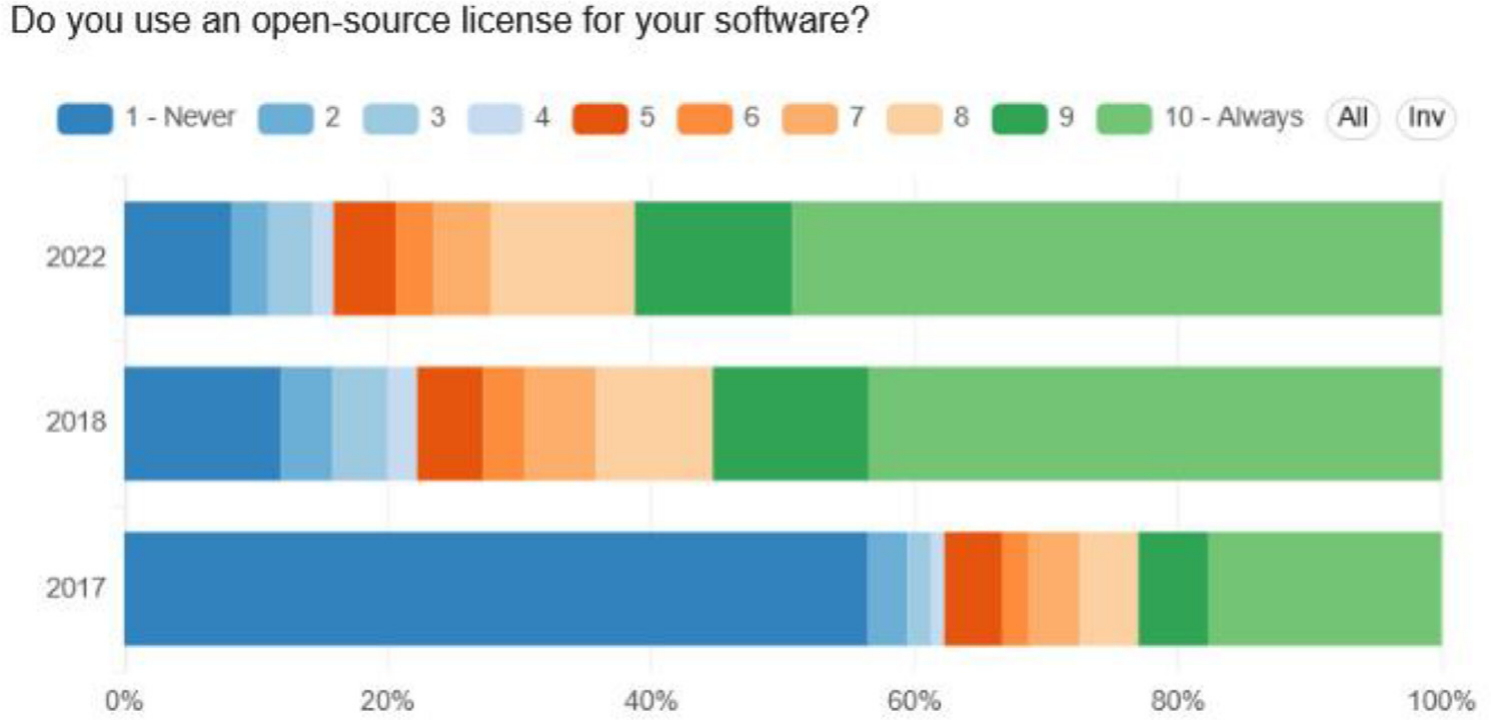
Answers to the question ``Do you use an open source license for your software?'' Question not included in the 2016 wave of the survey. [7].

**Fig. 3:**
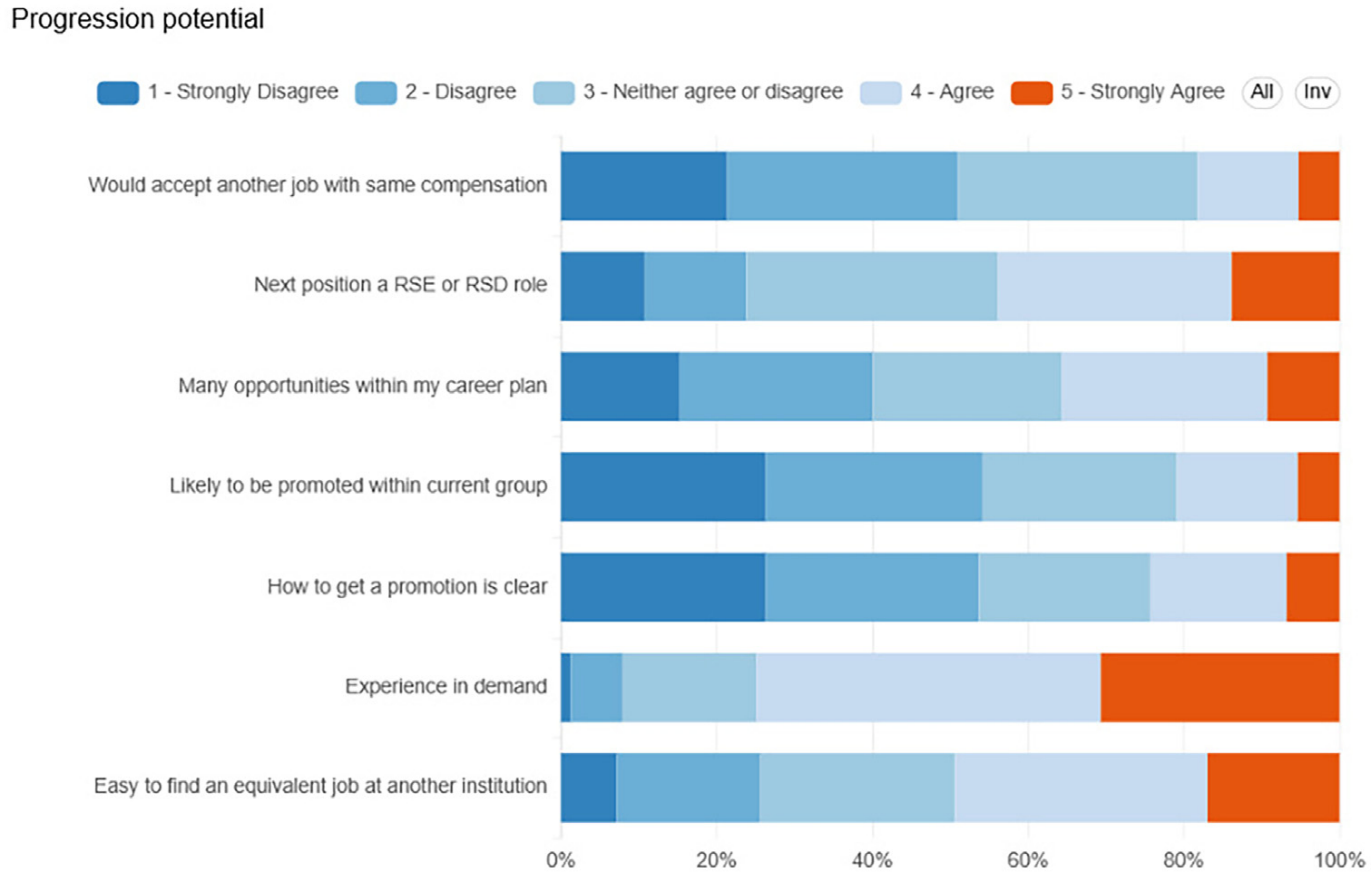
Answers to questions about career progression perspectives. [7].

**Fig. 4:**
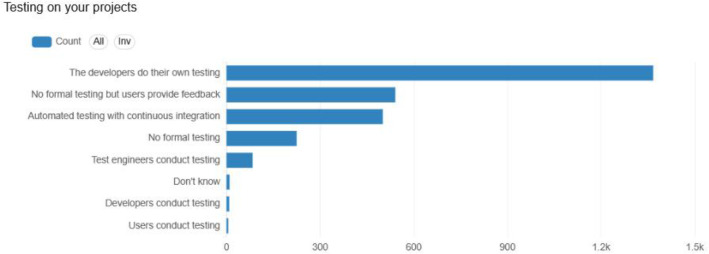
Answers to the question ``How do you test the software that you produce?'' [7].

**Fig. 5:**
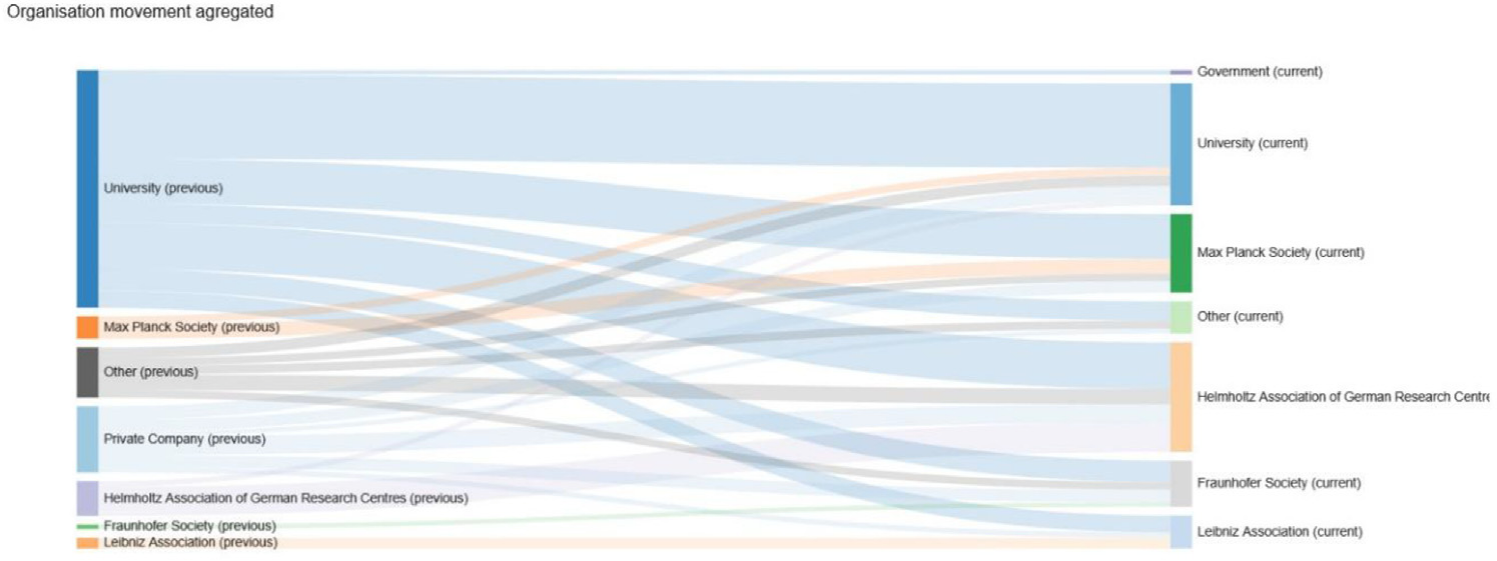
Answers to questions ``Where was your previous job based?'' and ``Please, select your organisation type.'' for Germany. [7].

**Fig. 6:**
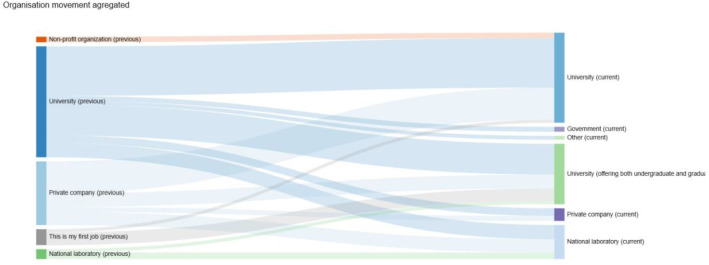
Answers to questions ``Where was your previous job based?'' and ``Please, select your organisation type.'' for US. [7].

Some examples of the data collected include the perception on whether one's research contribution is being recognised (Fig. 1), how often software produced is being released under an open-source license (Fig. 2), how the software is tested (Fig. 4), or how participants feel about their career progression perspectives (Fig. 3). The data can be separated to show results for a specific country, e.g. employment organisation-type movement in Germany (Fig. 5) or in the US (Fig. 6). All above figures come from the interactive dashboard mentioned above [7].

## Limitations

• Participation in all survey waves was voluntary, and no formal sampling frame was imposed. As a result, the data may be subject to self-selection bias, and response rates vary across countries and survey years.
• Not all countries participated in every survey wave, and some participating countries are represented by relatively small sample sizes. This may limit the robustness of cross-country comparisons and subgroup analyses for certain regions or time periods.
• Although the survey instrument maintained a unified core structure, minor country-level adaptations and translation differences may have influenced how specific questions were interpreted by respondents.
• All data are self-reported and may therefore be affected by reporting bias or inaccuracies, particularly for items related to job roles, skills, or working practices.
• Some demographic and contextual variables were not collected consistently across all survey years, which may constrain the scope of longitudinal analyses for specific research questions.
• Participation in the survey was anonymous, and no identifiable data was collected. This dataset must therefore be treated as repeated cross-sections.

## Ethics Statement

This work involved human subjects. Informed consent was obtained, the study followed the Declaration of Helsinki, and ethical approval was granted. Specifically, ethical governance for the survey series was provided through the University of Southampton. Publicly documented approvals include the 2016 UK survey (ERGO 18478), the 2017 UK survey (ERGO/FPSE/25269), the 2017 Germany survey (ERGO/FPSE/30440), the 2018 international survey (ERGO/FPSE/45232), and the 2021/22 international survey wave (ERGO 65619). Before public release, all response data has been anonymized to ensure that individual respondents could not be identified. The publicly released datasets for the 2016, 2017, 2018, and 2022 survey waves comprise anonymized responses accompanied by supporting documentation and are shared under open licences.

## CRediT Author Statement

**Wioleta Kijewska:** Data curation; Software; Validation; Visualisation; Writing – original draft; Writing – review and editing. **Heather S. Packer:** Funding acquisition; Methodology; Resources; Writing – original draft; Writing – review and editing; Supervision. **Simon Hettrick:** Conceptualisation; Funding acquisition; Methodology; Resources; Writing – review and editing; Supervision.

## Data Availability

ZenodoRSE_survey_longitudinal: RSE Survey longitudinal dataset up to 2022 (Original data).
